# PRESHA — Preventing severe hypertensive adverse events in pregnancy and childbirth: a study protocol for establishing a prospective cohort study and biobank in urban Tanzania

**DOI:** 10.3389/fgwh.2026.1812275

**Published:** 2026-07-08

**Authors:** Andrea Solnes Miltenburg, Richard Kiritta, Benson R. Kidenya, Hannah Brown Amoakoh, Peter C. J. I. Schielen, Felix Manyogote, Hanne Ochieng Lichtwarck, Albert Kihunrwa, Johanne Sundby, Ingvil Sørbye, Ewoud Schuit, Karel G. M. Moons, Anne Cathrine Staff, Elia Mmbaga, Joyce L. Browne

**Affiliations:** 1 Department of Community Medicine and Global Health, Institute of Health and Society, Faculty of Medicine, University of Oslo, Oslo, Norway; 2 Department of Obstetrics and Gynaecology, Catholic University of Health and Allied Sciences, Mwanza, Tanzania; 3 Department of Biochemistry and Molecular Biology, Catholic University of Health and Allied Sciences, Mwanza, Tanzania; 4 Noguchi Memorial Institute for Medical Research, University of Ghana, Accra, Ghana; 5 Julius Center for Health Sciences and Primary Care, University Medical Center Utrecht, Utrecht University, Utrecht, Netherlands; 6 Faculty of Medicine, Institute of Clinical Medicine, University of Oslo, Oslo, Norway; 7 Division of Obstetrics and Gynaecology, Oslo University Hospital, Oslo, Norway

**Keywords:** biobank, biomarkers, hypertensive disorders of pregnancy, maternal health, preeclampsia

## Abstract

**Objectives:**

Hypertensive disorders of pregnancy are a leading cause of maternal and perinatal mortality in low- and middle income settings. Yet, to date few large-scale prospective clinical studies in this field are conducted in sub-Saharan Africa. This paper describes the study protocol of a prospective cohort study, the PRESHA (PREventing Severe Hypertensive Adverse Events) cohort in Mwanza, Tanzania. The PRESHA study aims to reduce maternal and neonatal mortality and morbidity related to preeclampsia (PE) through improved prediction, prevention and clinical management. More specifically, within the cohort we aim to improve antenatal risk prediction of PE by externally validating existing biomarker-based (PlGF and sFlt-1) risk prediction models.

**Methods and design:**

Recruitment for a prospective cohort study of 3,000 women started August 2025 and is ongoing. Eligible for enrolment are women between 10 ^+^ ^0^ to 16 ^+^ ^0^ weeks of pregnancy at booking for antenatal care. Socio-demographic, environmental, clinical, health behaviours, resource use and care satisfaction data will be collected up to 12 weeks postpartum. Biological samples (serum, plasma, urine) will be collected at up to five moments: at 10 ^+^ ^0^ ≤ 15 ^+^ ^6^ weeks, 19 ^+^ ^0^ ≤ 23 ^+^ ^6^ weeks, 27 ^+^ ^0^ ≤ 31 ^+^ ^6^ weeks, upon diagnosis and at birth (including placenta biopsy). Main outcomes are confirmed diagnosis of PE and adverse maternal and perinatal outcomes. Biomarker analysis (sFlt-1 and PlGF) will commence after completed recruitment. We will establish gestational age (GA)-specific population reference values for these biomarkers, and describe the predictive accuracy of the biomarkers and biometric parameters for screening of PE. For existing prognostic models, we will evaluate the predictive performance in our cohort in terms of discrimination (area under the receiver operating characteristics curve) and calibration (calibration plot).

**Discussion:**

This prospective cohort study offers comprehensive and contextually relevant data contributing to improving antenatal risk prediction for PE in a low-resource setting. The establishment of a new pregnancy database in Tanzania allows for identification of the impact of social or environmental determinants of health, risks of pregnancy complications and effect of health seeking behaviour. Future discovery studies of other predictors and diagnostics tools for placenta disorders will be possible with the established biobank.

## Introduction

Hypertensive disorders in pregnancy (HDP) include pregnancy-induced hypertension, chronic hypertension, preeclampsia (PE) and eclampsia with associated increased maternal and perinatal morbidity and mortality. HDP affect up to 10% of all pregnancies, with PE affecting 3%–5% of pregnancies globally, causing 76,000 maternal deaths and 500,000 infant deaths annually (1, 2). HDP are currently a leading cause of maternal deaths in many countries in sub-Saharan Africa (SSA). PE is a complex multisystem disorder including high blood pressure and dysfunction of other organ systems. Left untreated, PE can result in eclampsia, characterized by seizures, stroke and, ultimately, death (3). Moreover, PE is associated with prematurity, fetal growth restriction, stillbirths and newborn mortality, as well as longterm morbidity for both the woman and the offspring. In the case of early-onset disease, expectant management to prolong the pregnancy is the aim, balancing the risk of offspring prematurity against maternal and offspring survival risks. Preterm PE (i.e., diagnosis <34 weeks gestation) is the subgroup of hypertensive disorder of pregnancy with the highest risk of placental dysfunction (4, 5) and highest maternal and offspring mortality and morbidity rates (6).

Interventions to prevent PE are enabled by accurate identification of women at the highest risk for PE. These pregnant women can receive risk-tailored clinical care with timely follow-up to reduce fatalities and morbidity. Primary prevention includes timely administration of low-dose aspirin (<16 weeks) for high-risk women identified during late first trimester (7). Secondary prevention starts after diagnosis of HDP/PE with close monitoring of blood pressure, organ function, maternal and fetal wellbeing and timely birth planning. Tertiary prevention is necessary to avoid further worsening or recurrence of complications, and includes administration of magnesiumsulphate in eclampsia or in women with severe PE features (8–10). Given the broad range of risk factors that can label women as ´high risk´, careful consideration of which risk groups are most likely to benefit is essential, especially in resource-limited settings.

Risk screening early in pregnancy is in many settings in SSA primarily based on clinical risk factors (e.g., prior HDP or chronic hypertension), which is neither specific nor sensitive (11, 12). Risk screening as pregnancy progresses may be based on other emerging clinical risk factors (e.g., anemia, gestational diabetes, urinary tract infection) as well as blood pressure and urine analysis for protein. A substantial body of evidence supports risk screening using various biomarkers, in particular Placental Growth Factor (PlGF) and soluble fms-like tyrosine kinase-1 (sFlt-1) (3). The sFlt-1/PlGF ratio or measurement of sFlt-1 or PlGF alone have been used in clinical trials to evaluate the usefulness of prediction of PE development (13, 14), assess severity (15) and rule-out PE in pregnancies with some PE-like features (16). Early PE risk prediction in gestational weeks 11–14 is most precise when PlGF levels are combined with clinical information and mean arterial pressure and ultrasound-evaluated blood flow of the uterine arteries (17).

A recent systematic review and meta-analysis on the use of biomarkers for screening identified that the predictive value of PlGF alone was not high enough for early diagnosis. However, the authors concluded that the mean difference in PlGF levels between PE and healthy pregnancies, appears to increase as the gestational age (GA) progresses (18). A recent study has shown that the sFlt-1/PlGF ratio is useful for discontinuation of low-dose aspirin treatment where a woman with a presumed high risk of PE (assessed in first trimester) has a low (≤38) sFlt-1/PlGF ratio in weeks 24–28. Some authors have also developed algorithms for how to act in the event of various test results (19). In addition to early risk screening, recent studies also showed utility for predicting severe forms of PE in patients with confirmed clinical diagnosis of PE (16), highly relevant in settings where premature delivery has large consequences due to lack of prematurity care resources. Pregnant women with a high sFlt-1/PlGF ratio more often had PE, preterm delivery, lower newborn weight, and a shorter time from testing to delivery (20).

While much is known about the pathophysiology of PE, associated co-variables, and prediction model development and performance, data from low- and middle-income countries (LMICs) is limited. Nearly all multivariable prediction models that include biomarkers to estimate an individual woman's risk of PE are only validated in high-income countries (21). Thus, the modifying role of social or environmental determinants of health, such as chronic parasitic infections (e.g., malaria) and other endemic infections on the impact of placenta disorders, omics diversity between populations from different ancestry (22), or the interaction between these remain understudied. For example, serum concentrations of PlGF and Sflt-1/PlGF ratio vary considerably with maternal characteristics and comorbidities. Studies from high-income and some LMIC settings show elevated levels in parous women, and in individuals of African, Afro-Caribbean, South Asian, and East Asian origin (23), and lower levels in women with obesity or insulin-dependent diabetes mellitus (24–26).

To investigate the effectiveness of risk estimation for PE in a LMIC country, taking into account the above considerations, we designed the PREventing Severe Hypertensive Adverse Events in pregnancy and childbirth (PRESHA) project. The PRESHA project is a collaborative research project between the Catholic University of Allied Sciences (CUHAS) in Tanzania, the University of Oslo (UIO) and Oslo University Hospital (OUS) in Norway, and the University Medical Center in Utrecht (UMCU) in the Netherlands. The overall aim of the PRESHA project is to reduce maternal and neonatal mortality and morbidity related to PE through improved prediction, prevention and clinical management. We employ a comprehensive, context-specific mixed-methods research approach in urban Mwanza in Tanzania. This paper describes a study protocol for one of the several research studies within the PRESHA project: the cohort study.

The aims of this prospective cohort are: 1) To external validate existing biomarker-based (PlGF and sFlt-1) PE risk prediction models, and explore the utility of adding biomarkers (PlGF and sFlt-1) to routine screening at antenatal care in a LMIC context. 2) To establish a pregnancy database and biobank in Mwanza, Tanzania. While biobanking is a vital component of clinical research internationally, sub-Saharan Africa (SSA) lacks well characterized biobanks, resulting in knowledge-gaps related to etiology, pathophysiological mechanisms and fewer locally relevant basic sciences discoveries that can be translated into novel diagnostics, prognostic or therapeutic strategies tailored to these regions (27).

## Methods and analysis

### Study design

**Figure 1:**
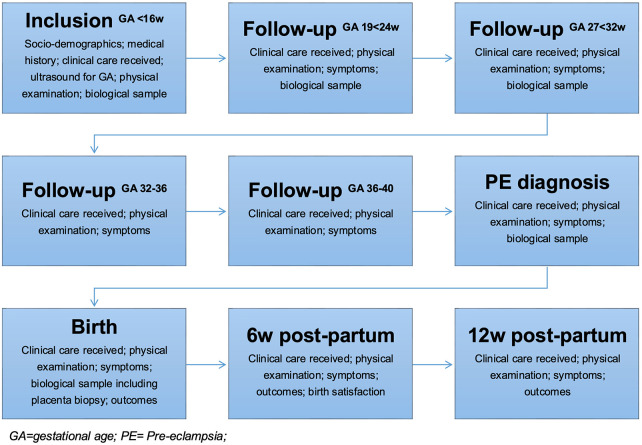
PRESHA study flowchart and data collection time points.

This PRESHA protocol describes a multi-center prospective cohort study including women of at least 16 years of age attending their first antenatal visit. Consecutive enrolment at participating facilities ensures a cohort representative of the target population in Tanzania. Prospective follow-up within facilities and across the referral chain is expected to minimize loss to follow-up and improve accurate detection of main outcome measurement, particularly for women who develop complications. Through this cohort we will also establish a pregnancy database and research biobank with samples collected at prespecified time points during pregnancy. A flowchart of the study and collection time points is presented in Figure 1. The study started including women in August 2025 and will continue to recruit new participants until September 2026, with expected follow-up to complete by June 2027.

### Study setting

The study will take place in Mwanza, the second largest city in Tanzania, located on the shores of Lake-Victoria. Mwanza city includes two districts, Nyamagana and Ilemela District, with a total population of nearly 1.2 million. The study sites for this study are located within both districts, representing a referral chain from primary to tertiary care: Nyamagana District Hospital, Buzuruga Health Center, Makongoro Health Center, Sekou-Toure Regional Hospital, and Bugando Medical Center (BMC). These public health facilities were purpousely selected because they cater for a large patient volume, appropriate infrastructure to conduct this study and staff willingness to participate.

In 2024, the annual number of women booking for antenatal visits was 24,109 and 28,241 at Ilemela and Nyamagana districts respectively, and among these women, 9,575 (18%) were attended to at the facilities where this study is conducted. In these districts a total number of 8,070 women were registered with a PE diagnosis in 2024, representing a registered PE rate of 15%.^1^ This high prevalence is likely due to high numbers of referrals from other districts to BMC. BMC is a tertiary referral center serving a population of 20 million people across eight regions. At this referral center, where the majority of severe PE cases are managed, PE contributes to an estimated 40% of maternal deaths and has a perinatal mortality rate of 20%.^2^ The associated PRESHA pregnancy database and biobank will be hosted by the Catholic University of Health and Allied Sciences/Bugando Medical Center (CUHAS/BMC).

### Recruitment and eligibility

Cohort participants will be recruited at Reproductive and Child Health (RCH) clinics at Nyamagana District Hospital, Buzuruga Health Center, Makongoro Health Center, and Bugando Medical Center. Makongoro Healh Center serves as the RCH clinic for Sekou-Toure Regional Hospital. Pregnant women attending RCH clinics for their antenatal care are informed about the study after being introduced to their routine antenatal programme as guided by the antenatal care clinic guidelines (10). To be eligible for inclusion in this study, a women must meet the following criteria: 1) attending their first antenatal visit and intending to deliver at one of the participating facilities, 2) GA 10 ^+^ ^0^≤15 ^+^ ^6^ determined by ultrasound measurement by trained sonographers, 3) age 16 years or above. The age limit of 16 reflects the legal age of marriage with parental consent in Tanzania, and that HDP/PE disproportionally affects young women. Women unable to understand oral or written information, unable to consent due to ill-health/illness or declining to participate will not be included.

### Main outcome measures

For the prospective cohort, the primary outcome measure is a PE diagnosis. Diagnosis of PE will be determined by new-onset hypertension accompanied by either significant proteinuria or other signs of maternal organ or uteroplacental dysfunction after 20 weeks of gestation, according to the 2018 International Society for the Study of Hypertension in Pregnancy (ISSHP) criteria (9). Secondary outcomes include onset of disease, disease severity (presence of severe features) and PE-related maternal and perinatal adverse outcomes. Maternal outcomes will include maternal death and maternal near-miss (based on sub-Saharan Africa Maternal Near Miss tool (28)). Perinatal adverse outcomes will include intrauterine or neonatal death and neonatal near miss (pragmatic markers to include birth weight <1750g, GA <33 weeks, Apgar score <7 at 5 min (29)).

### Sample size calculations and considerations

Sample size calculations are based on three scenarios; validation og existing models, new model development and a nested-case control study. We aim to recruite at least 3,000 participants, based on two power calculations with comparable outcomes. For validaton of known prediction models including early screening with PlGF alone, we assumed an event fraction for PE of 0.05 based on known prevalence rates for populations in SSA. We assume the distribution of the linear predictor to follow a normal distribution with a mean of −2.94 (corresponding to mean estimated risk of 5%) and a standard deviation of 0.8. This standard deviation corresponds to a lower 2.5% and upper 97.5% range of estimated risks of 1% and 20%, respectively. Furthermore, we assume a well-calibrated model with a calibration-in-the-large coefficient of 0, or similarly an observed/expected ratio of 1 and calibration slope of 1. The target confidence interval widths for these parameters, we assumed to be 0.4, which corresponds to an observed/expected ratio between 0.8 and 1.2 and a slope between 0.8 and 1.2. We assume a c-statistic of 0.7 with a confidence interval width of 0.1, corresponding to a c-statistic between 0.65 and 0.75. Based on these assumptions, and using the pmvalsampsize function from Riley et al. (30), we require a minimum sample size of 2,871 women for external prediction model validation. Because the required sample size is very sensitive to the assumed event fraction, and some loss to follow-up may be expected, the goal is to recruit at least 3,000 women.

We anticipate that if the performance of the existing prediction models is insufficiently accurate within the Tanzanian context, we will update existing models, e.g., by adjusting the intercept, slope or refitting predictor-outcome assocations, or even develop a new prediction model. This model will include the predictors: GA at presentation with symptoms, maternal age, parity, body-mass-index, PE diagnosis in one of previous pregnancies, PlGF, and sFlt-1. We use sample size criteria for development studies (31) to determine the required sample size for this scenario. If we allow for some modelling of functional forms additional to the seven predictors and assume 10 predictor parameters, an Nagelkerke's R-squared of 0.15, shrinkage of 0.9, an event fraction for PE of 0.05, a margin of error of 0.05, and a mean absolute prediction error of 0.05, the minimum sample size required for new model development is 1,787 (corresponding to 90 expected PE events and 8.94 events per predictor parameter). This sample size calculation in this scenario is well within the target of 3,000 women.

We also calculated the sample size for a nested case control study, for validation of risk-prediction models including measurement of PlGF and/or sFlt-1 later in pregnancy. Considering the 1:3 nested case-control study setting, i.e., a PE event fraction of 0.25, the linear predictor normal distribution with a mean of −1.10 (corresponding to a mean estimated risk of 25%) and a standard deviation of 0.8, and following the same assumptions as described above, we require a nested case-control population of 1,056 women (264 cases and 792 controls) to allow external validation of existing models that include relevant biomarkers. In the case of model development using a nested-case control (1:3) design, and following the same assumptions as described above, we require a nested case-control population of 844 women (211 cases and 644 controls) to allow the development of models that include relevant biomarkers. As all sample size calculations above are associated with significant uncertainties, a re-assessment of the number of participants needed to include is foreseen when ±1,000 participants are included and outcomes measures have been obtained.

### Study procedures

After consent, participants with a confirmed gestation 10 ^+^ ^0^ ≤ 15 ^+^ ^6^ weeks are assigned a unique identification number. This identification number is consistently used on the antenatal care card, study logbooks, data registration and biological samples, as taken during visits. Further details on sample collection, transport and storage can be found in the standard operating procedures (Supplementary Material 1). Women will be reminded of their follow-up visits by the study team through phone calls. The participant and facility staff are requested to contact the study team in the event of admissions or referrals. Data will be collected by trained clinical research assistants. Survey questions will be asked in Kiswahili, the local language. For data entry, project owned android tablets are used, using standardized data extraction forms in RedCap (version 13.11.4).

### Clinical data and biological sample collection

Data collected on socio-demographic, contextual, environmental, clinical and healthcare use variables are summarized in Table 1. The data and biological samples collected adhere to the proposed standardization of PE research, adapted to what is feasible within the context in Tanzania (32). Data collection will occur by administering questionnaires, and extraction from the antenatal card and other medical charts at the facility. Social determinants are defined according to the WHO as ´the conditions and environments in which people are born, live, work´ (33). Socio-demographics and contextual data include information on demographics and socio-economic-status that are largely based on a shortened version of the Tanzanian Demographic and Health Survey (TDHS) questions (34), including the wealth index, food security survey (35, 36), social support survey (37, 38) and questions related to environmental exposures. Within the cohort study, care experiences will be assessed through postpartum follow-up questionnaire, including the assessment of birth satisfaction (39).

**Table 1:** Study variables.

Variables at inclusion and follow-up	Variables during antenatal admissions and diagnosis	Outcome variables	Postpartum variables
Social and structural demographics ANC card/book number Address Contact information Age Country of birth Mariatal status Highest level of educaton Employment status Partners highest level of education Partners employment status Whealth index Perceived social support Environmental exposures Food security Medical history HIV status History of TB History of hemoglobinopathy diagnosis History of chronic condition History of blood transufions Family history of CVD Substance use Medication use Obstetric history Gravidity Parity Living children Number of previous abortions Number of previous intrauterine fetal deaths Number of previous early newborn deaths Contraceptive use Number of previous HDP Number of previous Gestational Diabetes Mellitus (DM) Number of previous premature births Number of previous births with low-birth weight Number of previous PPH Number of previous CS Mode of conception index pregnancy Last menstrual period GA by ultrasound Number of fetus Duration of sexual relationship with father of current pregnancy (prior to this pregnancy) Clinical care Height at booking Weight for each visit Blood pressure at each visit(diastolic/systolic) HIV/Syphilis status Hemoglobin (Hb) Proteinuria Fundal height Fetal heart rate Care use outside of ANC Informal care use OPD card number (if any) Identified risk factors during ANC Blood/urine samples taken for storage Symptoms Headache Blurred vision Nausea Difficulty breathing Tiredness Rapid or Irreguler heartbeat Difficulty sleeping Chest pain Reduction in urine output Vomiting Yellowness of the eyes Upper abdominal pain Swelling feet/face/vulva Confusion Numbness in extremities Fetal movements	Admission details Hospital file number Referral Number of admission days Blood/urine samples taken at diagnosis for storage Medication use before admission Date of discharge Medication at discharge Symptoms at diagnosis Headache Blurred vision Nausea Difficulty breathing Tiredness Rapid or Irreguler heartbeat Difficulty sleeping Chest pain Reduction in urine output Vomiting Yellowness of the eyes Upper abdominal pain Swelling feet/face/vulva Confusion Numbness in extremities Reduced fetal movements Clinical signs at diagnosis Highest blood pressure (BP) Proteinuria Urea Creatinine Uric acid Hb Lymphocytes Blood platelets ALT/AST Bilirubin Albumin Glucose INR Bedside clotting Fibrinogen Abnormal fetal heart rate Ultrasound findings Daily hospital registration Highest BP Proteinuria Lowest oxygen saturation ICU care admission Antihypertensive medication Magnesium sulphate Corticosteroids Treatment provided Symptoms	Birth details Abortion Mode of onset of birth Indication of induction Mode of birth Indicaton of CS CS knifetime Place of birth Cervical dilatation on admission Mode of rupture of membranes Highest BP during birth Number of BP measured Fetal presentation Fetal monitoring performed Maternal risk factors identified Admission days antepartum Description of amniotic fluid Duration of active labour Medication provided Indication for oxytocin Date of discharge Medication at discharge Blood/urine samples taken at birth for storage Neonatal outcomes Number of children Live born Sex Birth weight APGAR score at 1 and 5 min Newborn outcome NICU admission Diagnosis at NICU admission Maternal outcomes Type of HDP/PE Severe features present Peripartum complications Alive at discharge Maternal Near Miss (SSA standards) Maternal potential life-threathening conditions Pulmonary edema Dyspnea New-onset cerebral or visual disturbances Signs of intracranial hemorrhage, ischemic events Severe hypertension (>160/90) Severe persistent epigastric/right upper quadrant pain unresponsive to medication Sepsis Severe Postpartum Hemorrhage (>1000 mL) Eclampsia Uterine rupture Severe malaria Sickle cell crisis Elevated liver enzymes (AST/ALT ≥2 × upper limit) Thrombocytopenia (<100 × 10⁹/L) Progressive renal insufficiency (serum creatinine >1.1 mg/dL or doubling from baseline) Diffuse Intravascular Coagulation (DIC) ICU/HDU admission Intubation and ventilation (Not CS related) Hysterectomy IV hypertensives Provision of MgSO4 as treatment Biomarker readings at different GA timepoints, diagnosis and birth PlGF sflt-1 Placenta outcomes Placenta sample taken Weight of placenta	Birth and outcome details *(Patient perspectives)* Mode of start of birth Indication of induction Mode of birth Indication of CS Anesthesia used Referral Birth assistant Birth companion Respectful care Duration of stay at labour room Duration of admission Experience of complications Medication use HDP/PE diagnosis Newborn outcome APGAR score 1 and 5 min Birth weight Newborn admission to NICU Cause of death if fetal demise/neonatal death Postpartum care (6 and 12 weeks) Mother alive Newborn alive Initiation and contunued breastfeeding Mode of feeding Post partum care visits Highest recorded Blood pressure Readmissions Indication of readmission Medication use Duration of admission Birth satisfaction Final HDP diagnosis

Biological samples (i.e., serum, plasma, urine) will be collected at the same time as routine standard blood tests at the booking visit, and additional investigations specifically for this study. Samples will be collected according to local laboratory protocols and/or study protocols for tests not part of routine care (Table 2). Placenta tissue biopsies will be sampled following delivery, in line with international placenta sampling recommendations (40). Clinical data collected by the study team, in addition to routine care, include ultrasound for confirmation of GA, blood pressure measurement and urine analysis. In case of blood pressure levels ≥140 mmHg systolic and/or ≥90 mmHg diastolic, on repeat measurement, and urine dipstick results of asymptomatic bacteriuria, glucosuria or proteinuria (>1+), women will be referred to the local clinicians for further assessment. Women will be informed of the results of the ultrasound, blood samples (e.g., Hb levels) and results of urinary analysis for routine care, where applicable, and this will be documented on the antenatal card. Biological samples are stored in a biobank at BMC hospital. Samples will only be thawed immediately prior to analysis of biomarkers; biomarker analysis to commence when all recruitment is completed, possibly after completion of most deliveries.

**Table 2:** Biological sample collection and processing.

Type of sample	Sampling	Volume collected	Processing for storage
Urine	Inclusion (10 ^+^ ^0^ ≤ 15 ^+^ ^6^), and follow-up visits (19 ^+^ ^0^ ≤ 23 ^+^ ^6^ weeks, 27 ^+^ ^0^ ≤ 31 ^+^ ^6^), at PE diagnosis and birth/or at term and birth (non-PE)	30 mL of urine, immediate urineanalysis at collection site with URINOX-10 strip.	Two cryotubes of 1.5 mL of urine in 1.8 mL size cryotubes
Blood	Inclusion (10 ^+^ ^0^ ≤ 15 ^+^ ^6^), and follow-up visit (19 ^+^ ^0^ ≤ 23 ^+^ ^6^ weeks, 27 ^+^ ^0^ ≤ 31 ^+^ ^6^), at PE diagnosis and birth/or at term and birth (non-PE)	Three EDTA blood collection tubes and 1 serum blood collection tube, with 4 mL in each tube.	Three cryotubes of 1.5 mL of plasma and two cryotubes of 1.5 mL of serum in 1.8 mL size cryotubes
Placenta: maternal surface	Birth	One 2,5 cm deep tissue biopsy of maternal side	Sample storage in 1.8 mL size cryotube

For women diagnosed with or suspected of having HDP/PE, data will be collected from the medical records including clinical notes and findings of investigations at out-patient consultations, during admissions, and upon delivery. All daily measurements and treatment adaptations will be recorded. For women who do not develop PE or gestational hypertension, outcome measures will be obtained after birth. If women are referred to higher level care, data collection will continue at the next facility, as referral facilities are part of the study sites.

### Laboratory biochemical analysis of biomarkers

Blood samples will be analysed for PlGF and sFlt-1 biomarkers, using a standardised platform (e.g., Delfia Xpress or Roche Elecsys). Biomarkers will be analysed by laboratory personell in Mwanza. Samples will be blinded for PE outcome. PlGF and sFlt-1 biomarker test results will be stratified by HDP/PE status post analysis. PlGF and sFlt-1 results are considered both as continuous values using Multiple of the Median (MoM) values and dichotomous (into positive and negative test). As there is a known difference in concentration curves between women from different ancestries, and higher values have been observed in women with origin from SSA (41), we will compare biomarker concentrations of included women with global estimates and decide on the appropriateness to use pre-established cut off values. If the difference is too large, population-specific cut off values and median concentrations per gestational week will be established.

### Descriptive statistical analysis and prognostic modelling

We will first summarize baseline characteristics (means/SDs or medians/IQRs for continuous variables; percentages for categorical variables), and compare characteristics between women who develop PE and those who do not. PE incidence and timing of onset will be categorized based on GA at diagnosis. Kaplan–Meier survival curves will be used to analyse time from GA at booking to PE onset and time from PE diagnosis to delivery. Competing events (e.g., termination of pregnancy prior to PE development) will be accounted for using cumulative incidence functions. Regression analysis will assess the association between timing of PE onset (early or late onset PE or pre-term or term PE vs. no PE) and maternal and fetal outcomes, unadjusted or adjusted to known confounders. In multivariable analyses we will adjust for routine clinical and laboratory covariates (for example maternal age, parity, BMI, mean arterial pressure, and renal function markers). Univariate analysis will compare socio-demographic and clinical characteristics to the occurrence of PE compared to non-PE status. Multivariate logistic regression will be used to identify independent predictors of PE, in the case of new model development. All statistical analysis will be done using SPSS, STATA or R.

We will externally validate existing prognostic models for PE and report this according to the Transparent Reporting of multivariable prediction models for Individual Prognosis Or Diagnosis (TRIPOD) guidance (42). Existing prognostic models will be identified based on a reproducable literature search that includes PlGF and/or sFlt-1 as a predictor (43, 44), for which other predictors are measured within the PRESHA cohort, and that are ranked best in terms of risk of bias according to PROBAST (45). Preliminary prediction models identified are listed in Supplementary Material 2. An updated search will be done prior to analysis. We will validate each model per original specification for its predictive accuracy by estimating its discrimination (using the c-statistics/AUC with 95% CI), calibration (via calibration plots, calibration-in-the-large, and calibration slope), and decision curve analysis. To account for the nested case-control design controls will be weighed in the validation set with a sample fraction of 3.6, i.e., 2,850 non-PE women in the overall cohort divided by 792 controls in the nested study (46). When model performance at external validation is suboptimal, model updating in the validation population will be considered, i.e., recalibration-in-the-large, model recalibration, or model revision, as well as building a completely new model. In case of model building, state-of-the-art prediction model development and internal validation methodology will be applied. Missing data, both predictor and outcome data, will be addressed using multiple imputation by chained equations. If imputation is used, analyses will be performed on imputed datasets separately, and results will be pooled across multiply imputed datasets using Rubin's rules (47). To assess the incremental value of biomarker-based prediction, we will compare biomarker based PE prediction models to prediction models relying on clinical risk factors alone (mean arterial pressure and maternal risk factors).

### Data storage and access

The PRESHA project is a collaborative project across countries. A data protection impact assessment (DPIA) was performed to meet requirements in the General Data Protection Regulation (GDPR) Article 35. The data collection takes place in Tanzania and is CUHAS’ responsibility, as data controller. UIO and CUHAS are jointly responsible for storing and analysing data as shared data controllers, for the purpose of the PRESHA project objectives. Biological samples collected for the project will be stored in a biobank under the responsibility of CUHAS. The biobank at BMC is established under Tanzanian regulations. To protect participant confidentiality, personal identifiers are stored separately from research data: each participant is assigned a study ID in REDCap, the linkage key mapping study IDs to personal identifiers is accessible only to a small number of authorised project team members, and project datasets and biosample inventories contain only the study ID. End date for the processing of personal data in the PRESHA project will be 31.07.2033. After the end of the project, data is stored for documentation purposes in line with requirements from the ethical committee in Norway (REK), until 31.07.2038. All biological samples will be stored at BMC laboratory in Mwanza for up to 15 years after the end of the project. Storage will be done according to BMC protocols and procedures for storage in a −80 °C deep freezer. After data collection is completed for all participants, we will archive project generated data at the Services for sensitive data at University of Oslo, Norway (TSD), subject to a Data Transfer and Sharing Agreement as signed by partners. The PRESHA project is a member of the Global Pregnancy Collaboration (CoLab) initiative (https://pregnancycolab.tghn.org/). We intend to make data available upon reasonable request and in line with the informed consent given, after approval by the study consortium members and the appropriate ethics committees.

### Patient involvement

At the start of the project the PRESHA team has supported the establishment of a patient organization in Mwanza, Tanzania, Thrive beyond PreEclampsia Foundation (TPEF). TPEF is registered as a Community Based Organization within Mwanza. Members of TPEF have been consulted in the development and adjusments for the case record forms, pre-testing of the questionnaires and they offered input and advice for the consent forms and consenting procedures. TPEF is an important partner of PRESHA and their members are invited as co-researchers in the PRESHA project.

## Discussion

The PRESHA cohort study aims to improve antenatal risk prediction for PE early in pregnancy (late first trimester/early second trimester) by validating existing biomarker-based prediction models in SSA. We will also test the values of the angiogenic proteins sFlt-1 and PlGF as predictive and diagnostic parameters later in pregnancy. Pregnant women in LMIC, especially in SSA countries, like Tanzania, are disproportionally affected by serious maternal morbidity and mortality. Strategies to reduce the global burden of PE should therefore involve these settings. It is concerning that relatively few large-scale cohort studies and clinical trials including measurement of biomarkers have been conducted in LMIC; such studies are generally performed in high-income countries. Fortunately, a few important studies and trials are underway in Brazil, Gambia, Ghana, Kenya, Mosambique, Sierrra Leone, South Africa and Zambia (e.g., PAPAGAIO (48), PEARLS (49, 50), PRECISE (51), PREPARE (52), and PROVE (40)), but additional prospective cohort studies are still needed. Our study is the first cohort study with an associated biobank in a LMIC with recruitment of women below a GA of 16 weeks, with comprehensive clinical data and biological sample collection at up to five timepoints in pregnancy, with postpartum follow-up.

In this study our primary focus is on early pregnancy risk prediction, as low-dose aspirin prophylaxis to prevent such risks has been proven effective only when started below 16 weeks of gestation. We hypothesize that routine measurement of biomarkers during ANC visits will have good screening ability to predict the onset of preterm PE, independent of other possible prognostic factors. Adding biomarkers to existing prognostic models will improve predictive performance for asymptomatic women. Nevertheless, we recognize there are important barriers to implementing first trimester screening for PE in LMIC, utilizing the available algorithms (53). These complex algorithms typically assume routine first-trimester antenatal care attendance, ready access to ultrasound and advanced laboratory testing, clinicians trained to obtain and interpret results and availability of guidelines, follow-up and treatment options for those women identified as high risk. This is why in parallel, we expect to identify barriers and facilitators for risk prediction, prevention and management of HDP/PE within the study setting, and its potential implication if implemented. Findings from the cohort study and formative work will inform the design of the PRESHA trial, which aims to implement a package of interventions that integrates biomarker-based risk prediction, early initiation of low-dose aspirin for high risk women and targeted follow-up care.

Importantly, there are also emerging opportunities that can support successful early screening. Although care seeking for antenatal care in many LMICs was previously delayed until well in the second trimester, population data indicates increasing numbers of women now seek care earlier in pregnancy, creating opportunity for early initation of preventative measures (34). The World Health Organization recommends that all women receive at least one ultrasound during antenatal care (10), and the capacity of medical facilities for routine laboratory diagnostics has substantially improved over recent decades, driven in part by the scale-up of testing for other diseases (e.g., for malaria, HIV and COVID-19). These trends have increased the availability, acceptability and affordability of a testing infrastructure that can be repurposed for antenatal screening for HDP/PE. At the same time, rising clinic attendance and persistent human resource constrains in many urban areas of SSA underline the pressing need for tools that can support health providers to more accurately triage and predict which women are in need of higher level care or tailored care.

Through the PRESHA study and the establishment of a new pregnancy database and associated biobank in Tanzania we will expand our understanding of how social and environmental determinants of health, health seeking behaviour and health system factors influence the risk and outcomes of pregnancy complications. The study will also document how HDP/PE is currently diagnosed and managed within the setting and how referral pathways operate in practice. Identifying women at high risk for HDP/PE will only reduce mortality and morbidity if concomitantly improvements occur in the quality of care and with a functional referral system.

An expected challenge for our cohort study is the partial reliance on routine clinical records which may incompletely or inaccurately reflect care and lead to missing or misclassified data. To protect our primary outcome, key clinical measurements are generated by the study team (for example ultrasound-based GA assesment, regular blood pressure measurement, urine analysis for proteins and systematic symptom screening). Our presence at the health facilities may also alter provider behaviour and increase workload in already busy clinics. Some of these challenges are mitigated by continuous presence of research assistants, close collaboration with health providers and appropriate compensations for provider time. Some project-delivered services (including ultrasound, laboratory testing and structured follow-up) could themselves influence outcomes, but these activities are necessary to ensure high-quality, comparable data for the pregnancy database and biobank.

Beyond the immediate aims of the current study, the PRESHA pregnancy database and biobank will offer a durable platform for discovery and translational research, urgently needed in LMIC settings. Locally generated, well-annotated samples and linked clinical data will improve the validity and applicability of future research and will support the development of targeted interventions, that reflect the genetic, environmental and health system context in Tanzania, pending funding opportunities. Hosting the biobank in Tanzania ensures representation of the Tanzanian population in future studies and enables equitable sharing of benefits through joint governance, local leadership, capacity strengthening and importantly, context appropriate returning of results to the local community. The infrastructure generated will create unique opportunities for LMIC-led nested studies and through the high-quality standardized collection of data and biological samples and transparent data-sharing procedures, the PRESHA study will allow for future pooled analysis of larger datasets.

## Footnotes

^1^Ministry of Health, Regional Mwanza Office. Regional Reproductive and child health year report [unpublished] (2024).

^2^Bugando Medical Center. Maternal and Newborn health outcomes 2024. Bugando Medical Centre 2024 [unpublished] (2024).
